# Microencapsulated octreotide pamoate in advanced gastrointestinal and pancreatic cancer: a phase I study.

**DOI:** 10.1038/bjc.1998.435

**Published:** 1998-07

**Authors:** S. I. Helle, J. Geisler, J. P. Poulsen, K. Hestdal, K. Meadows, W. Collins, K. M. Tveit, J. M. Holly, P. E. Lønning

**Affiliations:** Department of Oncology, Haukeland University Hospital, Bergen, Norway.

## Abstract

**Images:**


British Journal of Cancer (1998) 78(1), 14–20
© 1998 Cancer Research Campaign

### Microencapsulated octreotide pamoate in advanced gastrointestinal and pancreatic cancer: a phase I study

SI Helle[1], J Geisler[1], JP Poulsen[2], K Hestdal[3], K Meadows[4], W Collins[5], KM Tveit[2], A Viste[6], JMP Holly[4] and PE Lønning[1]

[1]Department of Oncology, Haukeland University Hospital, N-5021 Bergen, Norway; [2]Department of Oncology, The Norwegian Radium Hospital, 0310 Oslo, Norway; [3]Novartis, Norway; [4]Department of Surgery, Bristol Royal Infirmary, Bristol, UK; [5]Novartis, Switzerland; [6]Department of Surgery, Haukeland University Hospital, N-5021, Bergen, Norway

**Summary** Fourteen patients suffering from advanced colorectal ($n$ = 7), pancreatic ($n$ = 4) or gastric ($n$ = 3) carcinomas received treatment with microencapsulated octreotide pamoate 90 mg i.m. every 4 weeks ($n$ = 4), 160 mg i.m. every 4 weeks ($n$ = 4) or 160 mg i.m. every 2 weeks ($n$ = 6). Two patients had stable disease, one for 4 and one for 6 months. Plasma insulin-like growth factor (IGF)-I decreased by 49–53%, IGF-II by 27–37% and total IGF-binding protein (IGFBP)-3 by 16–19%, whereas IGFBP-1 increased by 35–55%. Insulin and C-peptide levels decreased by 29–38% and 41–46% respectively. A non-significant decrease in urinary GH secretion and an increase in the ratio of fragmented to intact IGFBP-3 as well as IGFBP-3 protease activity was seen. The increase in IGFBP-3 fragmentation correlated negatively with alterations in IGF-I and IGF-II ($P < 0.05$). We conclude that microencapsulated octreotide administered in doses up to 160 mg every 2 weeks is well tolerated and has pronounced effects on several components of the IGF system in plasma. In addition, changes in IGFBP-3 protease activity because of cancer may contribute to alterations in IGF-I and -II, indicating the importance of measuring this parameter in addition to IGFs and IGFBPs when evaluating alterations in IGF-I.

**Keywords:** growth hormone; insulin-like growth factors; somatostatin

Advanced gastrointestinal and pancreatic cancers have a grave prognosis and pose serious problems in medical oncology. Current systemic treatment options are of limited value, and new strategies are needed to improve therapy. Somatostatin analogues are found to be useful in palliative treatment of certain endocrine gastrointestinal tumours (Schally, 1988). In vitro and animal studies have also shown somatostatin analogues to inhibit the growth of non-endocrine pancreatic and gastrointestinal tumours (Szepeshazi et al, 1991; Dy et al, 1992; Qin et al, 1992; Watson et al, 1992).

Although growth inhibition by somatostatin analogues may be a direct effect mediated by specific somatostatin receptors on tumour cells, such effects could also be achieved through inhibition of growth factors and growth stimulatory hormones. Somatostatin analogues inhibit secretion of gastrointestinal hormones with possible mitogenic effects (Adrian et al, 1981) and suppress synthesis of the insulin-like growth factor (IGF)-I (Pollak et al, 1989; Figg et al, 1995; Leo et al, 1995) by suppressing growth hormone (GH) secretion (del Pozo et al, 1986), the main trophic factor for IGF-I synthesis (Schwander et al, 1983). IGF-I and -II are important mitogens to many human cancer cell lines in vitro and altered bioavailability of these growth factors may potentially influence tumour growth (Macaulay, 1992).

Clinical studies evaluating the anti-tumour effect of somatostatin analogues in non-endocrine abdominal cancers have reported conflicting results (Savage et al, 1987; Klijn et al, 1990; Friess et al, 1993; Smith et al, 1994, Cascinu et al, 1995). A major obstacle to the development of somatostatin analogues in clinical use has been their short half-lives, demanding three daily injections (Kutz et al, 1986). In this study we evaluate the tolerability, clinical effects, pharmacokinetics and effects on plasma IGF-I and -II of a new microencapsulated formulation of octreotide pamoate in patients suffering from gastrointestinal and pancreatic carcinomas. Plasma IGFs are bound to specific IGF-binding proteins (IGFBPs) acting as carriers and also as modulators of IGF-I bioactivity in the tissues (Jones and Clemmons, 1995). Most IGF-I and -II circulate in a 150-kDa complex together with an acid-labile subunit (ALS) and IGFBP-3, and availability of these growth factors to the tissues may be affected by proteases acting on IGFBP-3 as substrate (Holly et al, 1993). Thus, apart from determining plasma IGF-I and -II together with IGFBP-1 and -3 with radioimmunoassay (RIA), we measured IGFBPs using Western ligand blotting and the functional status of IGFBP-3 using immunoblotting. In addition, we also measured IGFBP-3 protease activity in the plasma samples.

### PATIENTS AND METHODS

### Patients

Fourteen patients (eight men and six women) with progressive locally advanced and/or metastatic adenocarcinomas of abdominal origin were studied (Table 1). Any other systemic anti-cancer therapy had been terminated at least 4 weeks before inclusion in the study. All patients had a performance status (WHO) less than 2 and an expected survival of more than 3 months. None of the patients had any significant medical or surgical disorder apart

*Received 2 September 1997*
*Revised 15 December 1997*
*Accepted 29 December 1997*

*Correspondence to:* PE Lønning

Part of this work was presented at the 21st ESMO congress in Vienna 2–5 November 1996.

**Table 1** Clinical characteristics of the individual patients included in the study

| Patient number | Cohort | Primary tumour | Age (years) | BMI (kg m$^{-2}$) | Previous treatment | Metastatic location(s) |
|---|---|---|---|---|---|---|
| 1 | 1 | Pancreatic | 45 | 16.7 | None | Liver |
| 2 | 1 | Gastric | 79 | 24.6 | None | Pleura |
| 3 | 1 | Colorectal | 61 | 20.1 | Chemotherapy[a] | Lung |
| 4 | 1 | Colorectal | 74 | 28.8 | Chemotherapy[a] | Lung |
| 5 | 2 | Pancreatic | 63 | 24.7 | None | Locally advanced |
| 6 | 2 | Pancreatic | 71 | 16.3 | None | Liver, lymph nodes |
| 7 | 2 | Colorectal | 65 | 29.7 | Chemotherapy[a] | Liver |
| 8 | 2 | Colorectal | 51 | 22.3 | Chemotherapy[a] | Liver |
| 9 | 3 | Pancreatic | 54 | 23.7 | None | Liver |
| 10 | 3 | Gastric | 69 | 22.1 | None | Liver |
| 11 | 3 | Gastric | 68 | 14.4 | None | Liver |
| 12 | 3 | Colorectal | 57 | 24.8 | Chemotherapy[a] | Liver |
| 13 | 3 | Colotectal | 55 | 26.4 | Chemotherapy[a] | Lung |
| 14 | 3 | Colorectal | 66 | 22.5 | Chemotherapy[a] | Lung |

[a]5-Fluorouracil and leucovorin.

from their cancer. Median age was 64 years (range 45–79 years) and body mass index 22.8 kg m$^{-2}$ (range 14.4–29.7). The study was approved by the regional ethics committee. All patients gave their written informed consent to participate in the trial.

### Treatment schedule

Patients were assigned sequentially to one of three cohorts. The first cohort received octreotide pamoate (OncoLAR) 90 mg i.m. every 4 weeks, whereas cohorts 2 and 3 received octreotide 160 mg i.m. every 4 weeks and every 2 weeks respectively. Four to six patients were enrolled in each cohort, and the frequency of dose-limiting toxicities (grade 3) should be $\leq 1$ in four subjects or $\leq 2$ in six subjects to allow patients to be enrolled in the next cohort. Thus, four patients were treated in cohorts 1 and 2, whereas six patients were treated in cohort 3. Physical examination, evaluation of adverse effects and standard laboratory evaluations (haematology, chemistry and urine analysis) were performed weekly until first response evaluation at day 57. Response evaluation was carried out radiologically according to the UICC criteria. Patients showing either stable disease or an objective response were offered continuous treatment with octreotide pamoate in the same dose as they had received during the first 8 weeks of the study with weekly follow-up for toxicity. Patients in cohorts 1 and 2 not continuing maintenance therapy had follow-up visits every 4 weeks, whereas patients in cohort 3 had extended weekly examinations up to day 71 after commencing treatment and later every 4 weeks.

### Blood and urine sampling

Fasting blood samples for evaluation of the IGF system, insulin and C-peptide were obtained in heparinized vials on the morning of commencing treatment and subsequently on days 15, 29, 57, 71, 99, 127, 155 and 169 during the treatment period. Plasma was separated by centrifugation and stored at – 20°C until analysis. Overnight (12 h) urine was collected on the same days for estimation of GH secretion. Serum samples for measurement of octreotide levels were obtained weekly during the study period. At the day of drug administration additional samples were drawn before and 1, 2 and 3 h after injection.

### Materials

Human recombinant IGF-I and IGF-II were purchased from GroPep (Adelaide, Australia). Human recombinant non-glycosylated IGFBP-3 was a gift from Dr C Maack, Celtrix (Santa Clara, CA, USA). IGF-I, -II and IGFBP-3 were iodinated using the chloramine-T method. Labelled peptide was separated from non-incorporated $^{125}I$ using AcA 202 columns (BioSepra, Villeneuve, France) using 1 × 40 cm columns for IGF-I and -II and 1 × 10 cm columns for IGFBP-3.

### Assays

Plasma levels of IGF-I (Holly et al, 1988) and IGF-II (Davies et al, 1991) were measured using RIA after acid–acetone extraction. IGFBP-3 (Cwyfan-Hughes et al, 1993) and IGFBP-1 (Holly et al, 1988) and octreotide (del Pozo et al, 1986) were directly measured using RIA. Plasma insulin and C-peptide were measured using RIA kits purchased from Diagnostic Products Corporation (Los Angeles, CA, USA). Urinary GH was measured by a sensitive RIA kit obtained from BioMerieux (France) according to the manufacturer's instructions.

The IGFBP profile in the plasma was analysed by Western ligand blotting (WLB) using a modified version (Coulson et al, 1991) of the technique originally described by Hossenlopp (Hossenlopp et al, 1986). IGFBPs were visualized by autoradiography and quantified using a densitometric scanner (Pharmacia LKB, Uppsala, Sweden). The IGFBP pattern was compared with the profile of a normal plasma pool (NP), and samples from each patient were analysed in the same run for comparison.

After WLB the membranes were washed and blocked four times in 10 nM Tris-HCl (pH 7.4) containing 5% milk and 0.2% Tween 20. The membranes were then probed overnight with a polyclonal specific antiserum against IGFBP-3 purchased from Diagnostic Systems Laboratories (Webster, TX, USA) at a final dilution of 1:10000. The membranes were then developed using enhanced chemiluminescent reagents supplied by Amersham (Aylesbury, UK) according to the manufacturer's instructions and the films were subjected to densitometric scanning.

The IGFBP-3 proteolytic activity in plasma was examined using a modified version (Frost et al, 1993) of the technique described by Lamson et al (1991).

© Cancer Research Campaign 1998 

**Figure 1** Plasma levels of octreotide during treatment in the three cohorts given as mean levels with range. Only octreotide levels before injection are shown at days of drug administration. Values obtained 1, 2 and 3 h after injection are not included

### Statistics

In previous studies we found plasma levels of IGF-I and -II, and IGFBP-1 to be well fitted to a log normal distribution whereas IGFBP-3 was found to be normally distributed (Lønning et al, 1995; Helle et al, 1996). Thus, parameters are given as their geometrical mean value with 95% confidence intervals of the mean, with the exception of IGFBP-3 and plasma octreotide levels when the arithmetic mean values are given. Correlations between parameters were tested for using the Spearman rank correlation test.

### RESULTS

### Tolerability and clinical effects

Treatment with octreotide pamoate was well tolerated. Dose-limiting (grade 3) side-effects were not seen in any of the patients. The most common side-effect was diarrhoea (four events grade 1–2). One patient complained about dizziness, one experienced pain at the injection site and one female patient developed alopecia, possibly related to the medication. Owing to sustained diarrhoea (grade 2), one patient treated in cohort 3 terminated treatment on his own choice after one injection of octreotide only.

Eleven out of the 13 evaluable patients were found to have progressive disease on day 57 (the first response evaluation). One patient with pancreatic cancer (cohort 2) had stable disease up to day 85, whereas one patient with gastric cancer (cohort 3) had stable disease lasting for 6 months.

Although a slight weight loss was observed in most patients, only one patient lost more than 10% of his body weight during the study period. One patient had deteriorating liver function at response evaluation based on serum bilirubin levels (2.5 × upper normal range), whereas the other patients had serum levels within the normal range.

### Plasma octreotide levels

Mean plasma levels of octreotide during the treatment period are shown in Figure 1. Peak levels of octreotide at the first day of injection (data not shown) for all cohorts were generally two to five times higher (range 2.2–9.9) than plasma levels measured just before the next injection. This ratio decreased in all patients after repeated injections. The lowest plasma level of octreotide observed at any time during treatment was 3.4 ng $ml^{-1}$. No difference in plasma levels of octreotide between cohorts 1 and 2 was observed during the treatment period. However, steady-state levels of octreotide for cohorts 2 and 3 were not reached at day 57 when most patients terminated treatment. One patient in cohort 3 receiving long-term treatment (> 6 months) obtained his highest level (98.9 ng $ml^{-1}$) at day 113 with a subsequent gradual decline.

### Endocrine effects

Plasma levels of IGF-I, IGF-II, IGFBP-1, IGFBP-3 insulin and C-peptide measured using RIA before the first injection on day 1 and on treatment levels expressed as percentage of pretreatment values are shown in Table 2. No differences between the cohorts or diagnostic subgroups were observed for any parameter. Accordingly, all data were pooled for statistical analysis. The number of patients available to follow-up beyond day 57 was too small to permit any statistical analysis of these data.

© Cancer Research Campaign 1998

**Table 2** Plasma levels of IGF-I, IGF-II, IGFBP-1, IGFBP-3, insulin, C-peptide and urinary GH excretion at day 1 (before the first injection) and levels at different time intervals during treatment with octreotide pamoate given as a percentage of pretreatment levels. Values other than IGFBP-3 RIA are given as geometrical mean with 95% confidence intervals

| | **Measured values** | **Values given as % of pretreatment (day 1) values** | | |
|---|---|---|---|---|
| **Day** | **1** | **15** | **29**[a] | **57** |
| IGF-I (ng $ml^{-1}$) | 65 (49–85) | 51 (43–61) | 51 (38–66) | 46 (35–61) |
| IGF-II (ng $ml^{-1}$) | 275 (211–358) | 73 (64–83) | 69 (50–97) | 63 (48–83) |
| IGFBP-1 (ng $ml^{-1}$) | 41 (26–66) | 135 (101–181) | 150 (112–202) | 155 (95–252) |
| IGFBP-3 RIA (ng $ml^{-1}$) | 5293 (4560–6026) | 85 (77–93) | 84 (79–89) | 82 (77–93) |
| IGFBP-3 WLB[b] | 3.2 (1.3–7.6) | 66 (53–83) | 74 (54–98) | 51 (34–108) |
| IGFBP-3 fragments[c] | 0.36 (0.24–0.54) | 109 (88–135) | 98 (85–113) | 119 (85–166) |
| IGFBP-3 protease activity[d] | 113 (8–22) | 121 (85–172) | 127 (78–208) | 145 (88–240) |
| Insulin (mIU $ml^{-1}$) | 9.8 (7.2–13.3) | 71 (48–107) | 62 (46–87) | 62 (42–90) |
| C-Peptide (ng $ml^{-1}$) | 2.0 (1.4–2.9) | 58 (38–87) | 54 (42–70) | 59 (32–109) |
| GH (ng 12 h) | 8.3 (2.2–30.8) | 64 (25–162) | 35 (8–145) | 59 (24–167) |

[a]Two patients had blood samples obtained at day 22 instead of 29. Their values are included in the day 29 group. [b]Arbitrary units [c]Ratio of fragmented to total IGFBP-3. [d]Value given as percentage of control (day 1) samples with added protease inhibitor (EDTA).

**Figure 2** Western ligand blots (above) with corresponding immunoblots for IGFBP-3 (below) in samples from two patients before and during treatment with octreotide pamoate. The 42- to 44-kD band on the ligand blot corresponds to the two glycosylation forms of intact IGFBP-3, the 36-kDa and 24-kDa bands correspond to IGFBP-2 and IGFBP-4 respectively. The nature of the 30- to 32-kDa band was not established. Patient 1 had a rapid progressive disease and an increase in the ratio of fragmented to intact IGFBP-3, whereas patient 2 had a slower disease progression and small alterations in protease activity. NP, normal plasma

Plasma IGF-1 levels decreased by 49–54%, whereas IGF-II decreased by 27–37% at various time intervals on treatment. We also observed a moderate decrease (16–19%) in immunoreactive IGFBP-3. IGFBP-1 levels increased by 35–55%, whereas fasting levels of insulin and C-peptide decreased by 29–38% and 41–46% respectively. A negative correlation between alterations in plasma C-peptide or insulin and IGFBP-1 was observed at most time intervals, but except for the correlation between C-peptide and IGFBP-1 at day 15 ($P < 0.05$) none of these correlations was of statistical significance.

IGFBP-3 was evaluated by Western ligand blots and immunoblots in addition to RIA (Table 2). Densitometric scanning of ligand blots revealed a mean decrease in intact IGFBP-3 between 26% and 49%. This decrease was somewhat larger than that observed in total IGFBP-3 evaluated using RIA. Although we observed only a minor overall increase in the ratio of fragmented to intact IGFBP-3 and in IGFBP-3 protease activity, several patients had a substantially higher ratio of fragmented to intact IGFBP-3 in the samples obtained at the time when disease progression was recorded (Figure 2). A positive correlation between the decrease in IGF-I and -II plasma levels and increase in fragmented to intact IGFBP-3 was found at all time interval, but it was of statistical significance only on day 57 ($P < 0.05$). Densitometric scanning of low-molecular-weight IGFBPs on the

*© Cancer Research Campaign 1998* 

**Figure 3** IGF-I, IGF-II and IGFBP-3 levels measured using RIA in a patient receiving only one injection with octreotide before terminating treatment. The patient had a slow disease progression and minor changes in the IGFBP-3 protease activity until day 84

ligand blot did not reveal any alterations apart from a progressive (significant at days 29 and 57) increase from 19% to 40% in the 36-kDa band representing IGFBP-2 (data not shown).

One patient received only one injection of octreotide. In this patient all parameters returned to baseline levels at day 84 (Figure 3).

### DISCUSSION

Although previous somatostatin analogue formulations have been found to be generally well tolerated, two to three injections per day were required because of their short half-lives (Kutz et al, 1986). Plasma steady-state octreotide concentrations of 0.27–0.55 ng $ml^{-1}$ are reported to cause a 70–80% inhibition of arginine-stimulated growth hormone secretion in healthy humans (Marbach et al, 1992). However, much higher plasma levels of octreotide (about 500 ng $ml^{-1}$) have been reported to be necessary for significant in vivo growth inhibition of colon cancer xenografts in nude mice (Dy et al, 1992). The lowest value of octreotide measured in individual patients during treatment in our study was 3.4 ng $ml^{-1}$, whereas the mean levels for each cohort were considerably higher at different time intervals. Moderate variations in plasma levels and low peak concentrations suggest adequate depot function of i.m. administered octreotide pamoate. Although this suggests that all patients achieve plasma octreotide levels sufficient for effective GH suppression in healthy individuals, the conditions may be different in patients suffering from advanced cancer, as many patients have been reported to have elevated GH levels (Emermann et al, 1984; Klijn et al, 1990). The stepwise increase in mean plasma levels of octreotide in cohorts 2 (160 mg every 4 weeks) and 3 (160 mg every 2 weeks) until day 57 indicates that steady state was not reached before terminating drug administration in the majority of the patients. Owing to the continuous absorption from the depot formulation, and the fact that plasma drug availability after OncoLAR administration is not known, common pharmacokinetic variables such as terminal half-life, clearance rate and volume of distribution could not be determined in this study.

We found octreotide pamoate administered in doses up to 160 mg every second week to be well tolerated in patients with advanced cancer. The overall incidence of side-effects was low in all cohorts, and no grade 3 side-effects were observed.

Treatment with octreotide pamoate had pronounced effects on the IGF system. The 50% decrease in plasma levels of IGF-I is in accordance with recent reports on somatuline (Figg et al, 1995) and lanreotide (di Leo et al, 1995) as well as a previous study with octreotide (Pollak et al, 1989). Mean baseline levels of IGF-1 (65 ng $ml^{-1}$) and IGF-II (275 ng $ml^{-1}$) were much lower than the normal range of these peptides (IGF-1; 100–494 ng $ml^{-1}$ and IGF-II; 462–1042 ng $ml^{-1}$) used as reference by others (Lawrence et al, 1997). This may be explained partly by advanced age as well as weight loss in our patients, both known to decrease plasma IGF-I levels (Sara and Hall, 1990). It cannot be excluded that some of the further decrease in IGF-I and IGF-II observed beyond day 15 may be due to disease-related factors, but only a minority of patients experienced major weight loss or deteriorating liver function.

The drop in plasma IGF-I may be due to inhibition of GH secretion by octreotide causing decreased hepatic synthesis of this growth factor (Schwander et al, 1983), but other mechanisms may also operate (Serri et al, 1992). The observed 27–37% decrease in IGF-II levels was unexpected, as previous studies with the somatostatin analogue somatuline (Figg et al, 1995) as well as other hormonal therapies influencing IGF-I levels (Frost et al, 1996; Reed et al, 1992) were found to have no effects on IGF-II. We speculate that some of the decrease in IGF-II levels may be secondary to a GH-dependent decrease in IGFBP-3 in our patients. GH is the most important regulator for synthesis of ALS (Dai et al, 1994), which, together with IGFBP-3, is necessary for formation of the 150-kDa ternary complex. A decrease in ALS (not evaluated in our study) affecting formation of the ternary complex may subsequently reduce the amount of IGFBP-3 as well as available binding sites for IGFs in this complex. The possible explanation may be that low-molecular-weight complexes consisting of only IGF-I or -II and an IGF-binding protein (including IGFBP-3) have a shorter half-life than the ternary complex (Guler et al, 1989). The decrease in IGFBP-3 is probably not secondary to the fall in IGF-I, as a previous study has shown that although administration of GH increases plasma IGFBP-3 levels it was decreased by administration of IGF-I (Kupfer et al, 1992).

Although most of the observed alterations in IGF-I, -II and IGFBP-3 may be explained by effects of treatment with octreotide, an increase in IGFBP-3 protease activity related to disease progression may also influence these parameters. Although total IGFBP-3 determined using RIA decreased by 15–20% only, densitometric scanning of IGFBP-3 on WLB revealed a larger suppression of intact IGFBP-3 (26–49%). Most IGFBP-3 RIAs detect both intact IGFBP-3 as well as fragments, and the observed increase in IGFBP-3 protease activity is not reflected by the RIA results. Discrepancies between RIA and WLB have also been observed by others (Gargosky et al, 1992), indicating the importance of evaluating IGFBP-3 also by WLB and immunoblots. The decrease in IGF-I and -II was also positively correlated with an increase in IGFBP-3 fragmentation at day 57, indicating that alterations in IGFBP-3 protease activity may also influence plasma levels of IGF-I and -II. An increase in IGFBP-3 protease activity has been reported to increase plasma clearance in IGF-I in rats (Davenport et al, 1990). Alternatively, the increase in IGFBP-3 protease activity may have been a compensatory response to the fall in plasma IGF-I and IGF-II in order to maintain availability of

 © Cancer Research Campaign 1998

IGFs to the tissues. The IGFBP-3 protease activity has been reported to be high in GH-deficient patients and low in acromegalic patients, consistent with an inverse relationship with IGF-levels (Lassarre et al, 1994).

Our finding of an increase in fasting plasma levels of IGFBP-1 is in accordance with the findings of others (Ezzat et al, 1992; Wolthers et al, 1994). Plasma IGFBP-1 is inversely correlated with insulin levels in healthy subjects, and insulin is known to be one of the most important regulators of plasma IGFBP-1 (Holly et al, 1988). Data from some studies indicate an insulin-independent regulation of IGFBP-1 by somatostatin analogues (Ezzat et al, 1992; Wolthers et al, 1994). However, other investigators have reported an inverse correlation between insulin and IGFBP-1 also during treatment with octreotide (Fredstorp et al, 1994), and hyperinsulinaemia was found to abolish somatostatin-stimulated IGFBP-1 release (Ørskov et al, 1994). In this study, fasting plasma levels of both insulin and C-peptide were significantly decreased and correlated negatively with alterations in plasma levels of IGFBP-1. Thus, our data support a role of insulin in the regulation of IGFBP-1 during treatment with octreotide. It is difficult to assess the influence of an increase in IGFBP-1 on the biological actions of IGF-I as this binding protein may inhibit but also enhance IGF-I effects in vivo, depending on the phosphorylation status of this binding protein (Jyung et al, 1994).

Densitometric scanning of WLB revealed an increase in the 36-kDa band corresponding to IGFBP-2. In a previous study, IGFBP-2 was found to be increased in many cancer patients (Kanety et al, 1993), and it is possible that the increase in this binding protein observed during treatment may be associated with disease progression rather than any influence of drug treatment.

Somatostatin analogues are known to inhibit GH secretion (del Pozo et al, 1986). Surprisingly, we did not observe a consistent suppression of urinary GH in our patients. Urinary 12-h GH secretion was measured using a sensitive RIA, and previous studies have shown a good correlation between urinary GH secretion and plasma GH profile (Girard and Fischer-Wasels, 1990; Hourd and Edvards, 1989). However, only a small amount (< 0.01%) of plasma GH is normally excreted in the urine (Baumann and Abramson, 1983), and whether this fraction may change in patients with advanced cancer is not known. We observed particularly high values in the last urinary samples obtained in patients with rapid progressive disease. Thus, the validity of urinary GH measurements in patients suffering from advanced cancer may be questionable. But it is also possible that no clinically significant suppression of plasma GH is obtained by treatment with octreotide in these patients as has been observed by others (Klijn et al, 1990).

In conclusion, octreotide pamoate in doses up to 160 mg every second week provides high plasma drug levels and is well tolerated in patients with advanced cancer. All doses administered were found to significantly suppress plasma levels of IGF-I, IGF-II, IGFBP-3, insulin and C-peptide, and to increase plasma levels of IGFBP-1. Whether suppression of plasma IGF-I and -II may be of importance regarding tumour growth is not yet clear. The durable endocrine effects and clinical tolerance suggest that octreotide administered as its pamoate depot formulation should be evaluated in further trials in cancer patients.

### ACKNOWLEDGEMENTS

The technical assistance of Mr D Ekse is highly appreciated. We are grateful to Celtrix Pharmaceuticals for the generous provision of the recombinant IGFBP-3 used in the assays. We would also like to thank Dr Christophe Gerbeau (Pharmacokinetic Unit, Laboratoire Sandoz, Rueil Malmaisone, France) for performing the octreotide assays. This work was supported by grants from the Norwegian Cancer Society.

### REFERENCES

Adrian TE, Barnes AJ, Long RG, O'Shaughnessy DJ, Brown MR, Rivier JE, Vale W, Blackburn AM and Bloom SR (1981) The effects of somatostatin analogs on secretion of growth, pancreatic, and gastrointestinal hormones in man. *J Clin Endocrinol Metab* **53**: 675–681

Baumann G and Abramson EC (1983) Urinary growth hormone in man: evidence for multiple molecular forms. *J Clin Endocrinol Metab* **56**: 305–311

Cascinu S, Ferro ED and Catalano G (1995) A randomised trial of octreotide *vs* best supportive care only in advanced gastrointestinal cancer patients refractory to chemotherapy. *Br J Cancer* **71**: 97–101

Coulson VJ, Wass JAH, Abdulla AF, Cotterill AM and Holly JMP (1991) Insulin-like growth factor binding proteins (IGFBPs) in acromegaly. *Growth Reg* **1**: 119–124

Cwyfan-Hughes SC, Wass JAH and Holly JMP (1993) Two site-specific radioimmunoassays which demonstrate the presence of proteolytically modified insulin-like growth factor-binding protein-3 in circulation. *J Endocrinol* **137**: 321–328

Dai J, Scott CD and Baxter RC (1994) Regulation of the acid-labile subunit of the insulin-like growth factor complex in cultured rat hepatocytes. *Endocrinology* **135**: 1066–1072

Davenport ML, Clemmons DR, Miles MV, Camacho-Hubner C, D'Ercole AJ and Underwood LE (1990) Regulation of serum insulin-like growth factor-I (IGF-I) and IGF binding proteins during rat pregnancy. *Endocrinology* **127**: 1278–1286.

Davies SC, Wass JAH, Ross RJM, Cotterill AM, Buchanan CR, Coulson VJ and Holly JMP (1991) The induction of a specific protease for insulin-like growth factor binding protein-3 in the circulation during severe illness. *J Endocrinol* **130**: 469–473

del Pozo E, Neufeld M, Schlüter K, Tortosa E, Clarenbach P, Bieder E, Wendel L, Nüesch E, Marbach P, Cramer H and Kerp L (1986) Endocrine profile of a long-acting somatostatin derivative SMS 201-995. Study in normal volunteers following subcutaneous administration. *Acta Endocrinol* **111**: 433–439

di Leo A, Ferrari L, Bajetta E, Bartoli C, Vicario G, Moglia D, Miceli R, Callegari M and Bono A (1995) Biological and clinical evaluation of Lanreotide (BIM 23014), a somatostatin analogue in the treatment of advanced breast cancer. *Breast Cancer Res Treat* **34**: 237–244

Dy DY, Whitehead RH and Morris DL (1992) SMS 201.995 inhibits *in vitro* and *in vivo* growth of human colon cancer. *Cancer Res* **52**: 917–923

Emermann JT, Leahy M, Gout PW and Bruchowski N (1984) Elevated growth hormone in sera from breast cancer patients. *Hormon Metab Res* **17**: 421–424

Ezzat S, Ren S-G, Braunstein GD and Melmed S (1992) Octreotide stimulates insulin-like growth factor binding protein-1: a potential pituitary-independent mechanism for drug action. *J Clin Endocrinol Metab* **75**: 1459–1463

Figg WD, Thibault A, Cooper MR, Reid R, Headlee D, Dawson N, Kohler DR, Reed E and Sartor O (1995) A phase I study of the somatostatin analogue somatuline in patients with metastatic hormone-refractory prostate cancer. *Cancer* **75**: 2159–2164

Fredstorp L, Werner S, Bang P and Hall K (1994) Inverse correlation between insulin-like growth factor binding protein-1 and insulin in patients with acromegaly during treatment with the somatostatin analogue octreotide. *Clin Endocrinol* **41**: 495–501

Friess H, Büchler M, Beglinger C, Weber A, Kunz J, Fritsch K, Dennler HJ and Beger HG (1993) Low-dose octreotide treatment is not effective in patients with advanced pancreatic cancer. *Pancreas* **8**: 540–545

Frost VJ, Macaulay VM, Wass JAH and Holly JMP (1993) Proteolytic modification of insulin-like growth factor binding proteins: comparison of conditioned media from human cell lines, circulating proteases and characterized enzymes. *J Endocrinol* **138**: 545–554

Frost VJ, Helle SI, Lønning PE, van der Stappen JWJ and Holly JMP (1996) Effects of treatment with megestrol acetate, aminoglutethimide or formestane on insulin-like growth factor (IGF) I and II, IGF-binding proteins (IGFBPs) and IGFBP-3 protease status in patients with advanced breast cancer. *J Clin Endocrinol Metab* **81**: 2216–2221

Gargosky SE, Pham HM, Wilson KF, Liu F, Guidice LC and Rosenfeld RG (1992) Measurement and characterization of insulin-like growth factor binding protein-3 in human biological fluids: discrepancies between radioimmunoassay and ligand blotting. *J Clin Endocrinol Metab* **131**: 3051–3060

Girard J and Fischer-Wasels T (1990) Measurement of urinary growth hormone. *Hormon Res* **33** (suppl 4): 12–18

Guler H-P, Zapf J, Schmid C and Froesch ER (1989) Insulin-like growth factors I and II in healthy man. Estimation of half-lives and production rates. *Acta Endocrinol* **121**: 753–758

Helle SI, Holly JMP, Tally M, Hall K, van der Stappen J and Lønning PE (1996) Influence of treatment with tamoxifen and change in tumour burden on the IGF-system in breast cancer patients. *Int J Cancer* **69**: 335–339

Holly JMP, Biddlecombe RA, Dunger DB, Edge JA, Amiel SA, Howell R, Chard T and Rees LH (1988) Circadian variation of GH-independent IGF-binding protein in diabetes mellitus and its relationship to insulin. A new role for insulin. *Clin Endocrinol* **29**: 667–675

Holly JMP, Claffey DCP, Cwyfan-Hughes SC, Frost VJ and Yateman ME (1993) Proteases acting on IGFBPs: their occurrence and physiological significance. *Growth Regul* **3**: 88–91

Hossenlopp P, Seurin D, Segovia-Quinson B, Lassarre C, Hardouin S and Binoux M (1986) Analysis of serum insulin-like growth factor binding proteins using Western ligand blotting: use of the method of titration of the binding proteins in competitive binding studies. *Anal Biochem* **154**: 138–143

Hourd P and Edvards R (1989) Measurement of human growth hormone in urine: development and validation of a sensitive and specific assay. *J Endocrinol* **121**: 167–175

Jones JI and Clemmons DR (1995) Insulin-like growth factors and their binding proteins: biological actions. *Endocrine Rev* **16**: 3–34

Jyung RW, Mustoe TA, Busby WH and Clemmons DR (1994) Increased wound-breaking strength induced by insulin-like growth factor I in combination with insulin-like growth factor binding protein 1. *Surgery* **115**: 233–239

Kanety H, Madjar Y, Dagan Y, Levi J, Papa MZ, Pariente C, Goldwasser B and Karasik A (1993) Serum insulin-like growth factor-binding protein-2 (IGFBP-2) is increased and IGFBP-3 is decreased in patients with prostate cancer: correlation with serum prostate-specific antigen. *J Clin Endocrinol Metab* **77**: 229–233

Klijn JGM, Hoff AM, Planting AST, Verweij J, Kok T and Lamberts SWJ (1990) Treatment of patients with metastatic pancreatic and gastrointestinal tumours with the somatostatin analogue sandostatin: a phase II study including endocrine effects. *Br J Cancer* **62**: 627–630

Kupfer SR, Underwood LE, Baxter RC and Clemmons DR (1992) Enhancement of the anabolic effects of growth hormone and insulin-like growth factor I by use of both agents simultaneously. *J Clin Invest* **91**: 391–396

Kutz K, Nüesch E and Rosenthaler J (1986) Pharmacokinetics of SMS 201-995 in healthy subjects. *Scand J Gastroenterol* **21** (suppl. 119): 65–72

Lamson G, Guidice LC and Rosenfeld RG (1991) A simple assay for proteolysis of IGFBP-3. *J Clin Endocrinol Metab* **72**: 1391–1393

Lassarre C, Lalou C, Perin L and Binoux M (1994) Protease-induced alteration of insulin-like growth factor binding protein-3 as detected by radioimmunoassay. Agreement with ligand blotting data. *Growth Regul* **4**: 48–55.

Lawrence JB, Conover CA, Haddad TC, Ingle JN, Reid JM, Ames MM, Suman VJ, Marks RS, Erlichman C and Hartmann LC (1997) Evaluation of continuous infusion suramin in metastatic breast cancer: impact of plasma levels of insulin-like growth factors (IGFs) and IGF-binding proteins. *Clin Cancer Res* **3**: 1713–1720

Lønning PE, Helle SI, Johannessen DC, Adlercreutz H, Lien EA, Tally M, Ekse D, Fotsis T, Anker GB and Hall K (1995) Relations between sex hormones, sex hormone binding globulin, insulin-like growth factor-I and insulin-like growth factor binding protein-I in post-menopausal breast cancer patients. *Clin Endocrinol* **42**: 23–30.

Macaulay VM (1992) Insulin-like growth factors and cancer. *Br J Cancer* **65**: 311–320.

Marbach P, Briner U, Lemaire M, Schweitzer A and Terasaki T (1992) From somatostatin to Sandostatin: pharmacodynamics and pharmacokinetics. *Metabolism* **41** (suppl. 2): 7–10

Pollak MN, Polychronakos C and Guyda H (1989) Somatostatin analogue SMS 201–995 reduces serum IGF-I levels in patients with neoplasms potentially dependent on IGF-I. *Anticancer Res* **9**: 889–892

Qin Y, Schally AV and Willems G (1992) Treatment of liver metastases of human colon cancers in nude mice with somatostatin analogue RC-160. *Int J Cancer* **52**: 791–796

Reed MJ, Christodoulides A, Koistinen R, Säppelä M, Teale JD and Ghilchik MW (1992) The effect of endocrine therapy with medroxyprogesterone acetate, 4-hydroxyandrostenedione or tamoxifen on plasma concentrations of insulin-like growth factor (IGF)-I, IGF-II and IGFBP-1 in women with advanced breast cancer. *Int J Cancer* **52**: 208–212

Sara VR and Hall K (1990) Insulin-like growth factors and their binding proteins. *Physiol Rev* **70**: 591–614

Savage AP, Calam J, Wood CB and Bloom SR (1987) SMS 201-995 treatment and advanced intestinal cancer: a pilot study. *Aliment Pharmacol Therap* **1**: 133–139

Schally AV (1988) Oncological applications of somatostatin analogues. *Cancer Res* **48**: 6977–6985

Schwander JC, Hauri C, Zapf J and Froesch ER (1983) Synthesis and secretion of insulin-like growth factor and its binding protein by the perfused rat liver: dependence on growth hormone status. *Endocrinology* **113**: 297–305

Serri O, Brazeau P, Kachra Z and Posner B (1992) Octreotide inhibits insulin-like growth factor-I hepatic gene expression in the hypophysectomized rat: evidence for a direct and indirect mechanism of action. *Endocrinology* **130**: 1816–1821

Smith JP, Doll D, Croitoru R, Thornton C and Perry MC (1994) Octreotide has no effect on advanced colon cancer. *J Clin Gastroenterol* **18**: 245–247

Szepeshazi K, Schally AV, Cai R-Z, Radulovic S, Milovanovic S and Szoke B (1991) Inhibitory effect of bombesin/gastrin-releasing peptide antagonist RC-3095 and high dose of somatostatin analogue RC-160 on nitrosamine-induced pancreatic cancers in hamsters. *Cancer Res* **51**: 5980–5986

Watson SA, Morris DL, Durrant LG, Robertson JF and Hardcastle JD (1992) Inhibition of gastrin-stimulated growth of gastrointestinal tumour cells by octreotide and the gastrin/cholecystokinin antagonists, proglumide and lorglumide. *Eur J Cancer* **8**: 1462–1467

Wolthers T, Grøfte T, Flyvbjerg A, Frystyk J, Vilstrup H, Ørskov H and Foegh M (1994) Dose-dependent stimulation of insulin-like growth factor-binding protein-1 by lanreotide, a somatostatin analog. *J Clin Endocrinol Metab* **78**: 141–144

Ørskov H, Wolthers T, Grøfte T, Flyvbjerg A, Vilstrup H and Hamberg O (1994) Somatostatin-stimulated insulin-like growth factor binding protein-1 release is abolished by hyperinsulinemia. *J Clin Endocrinol Metab* **78**: 138–140

 © Cancer Research Campaign 1998


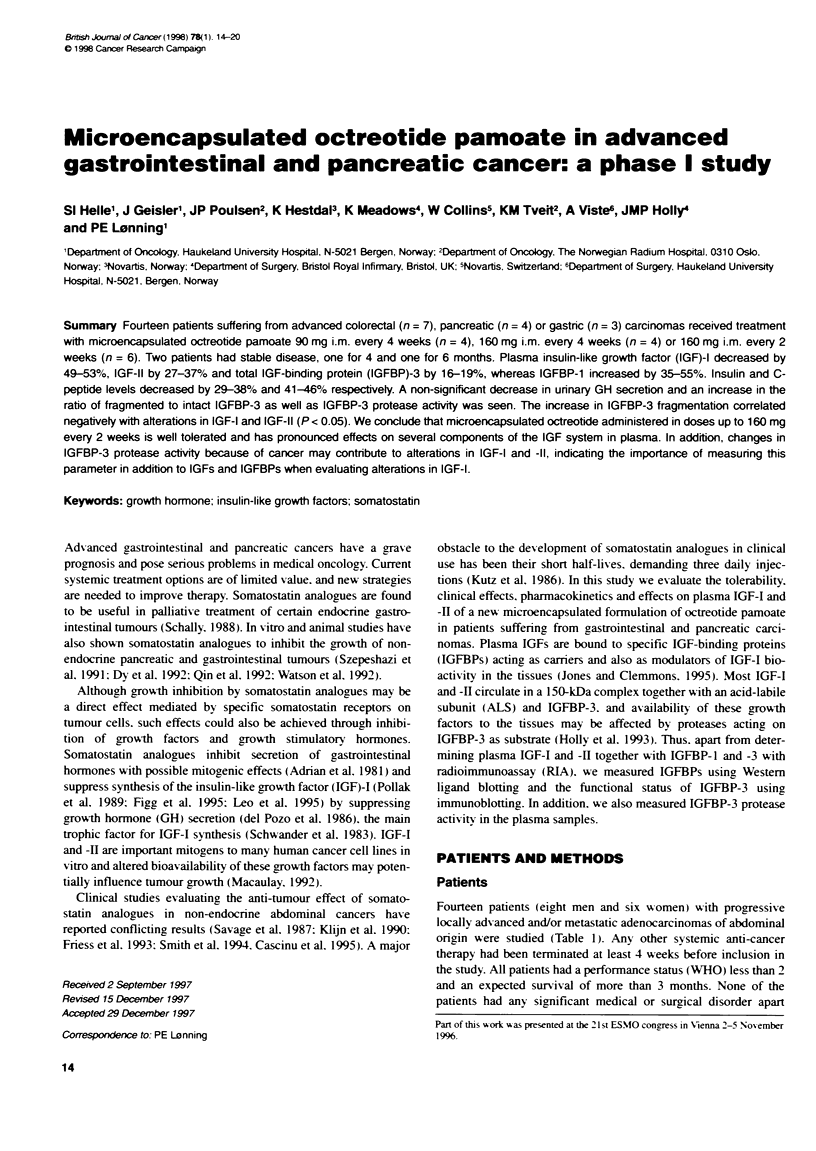

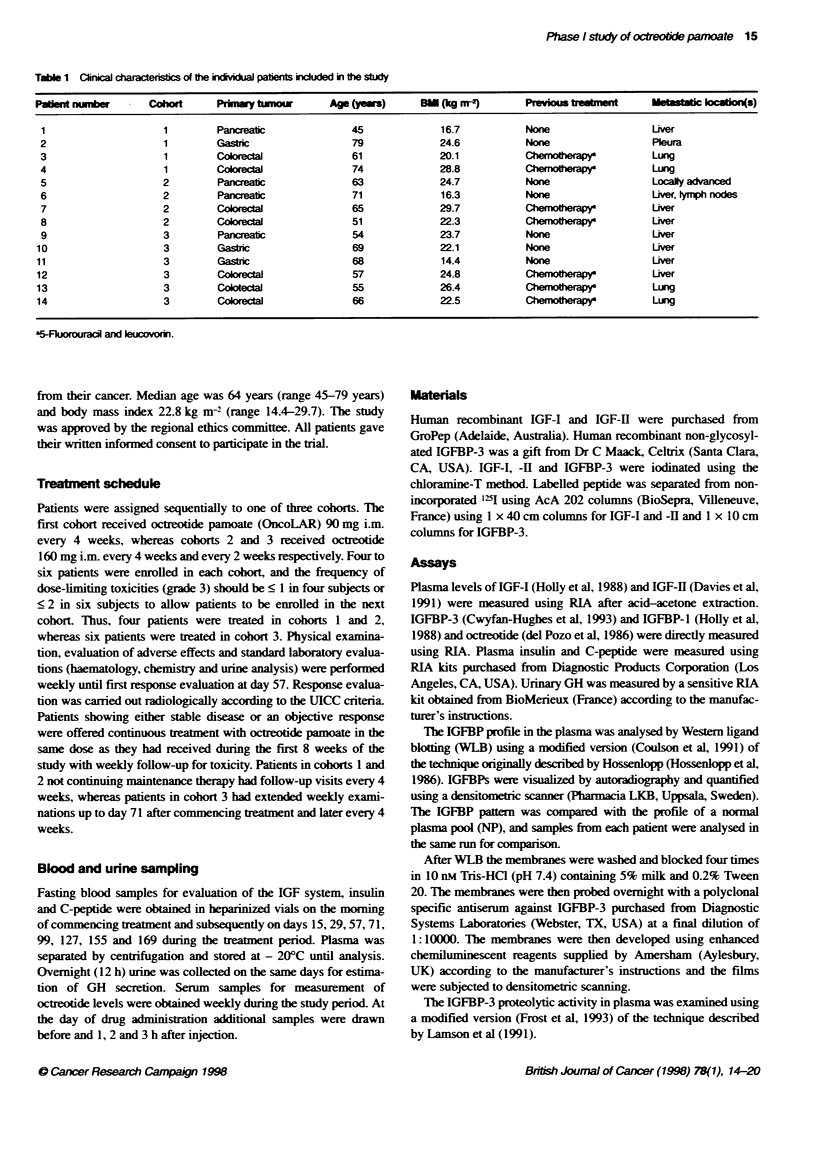

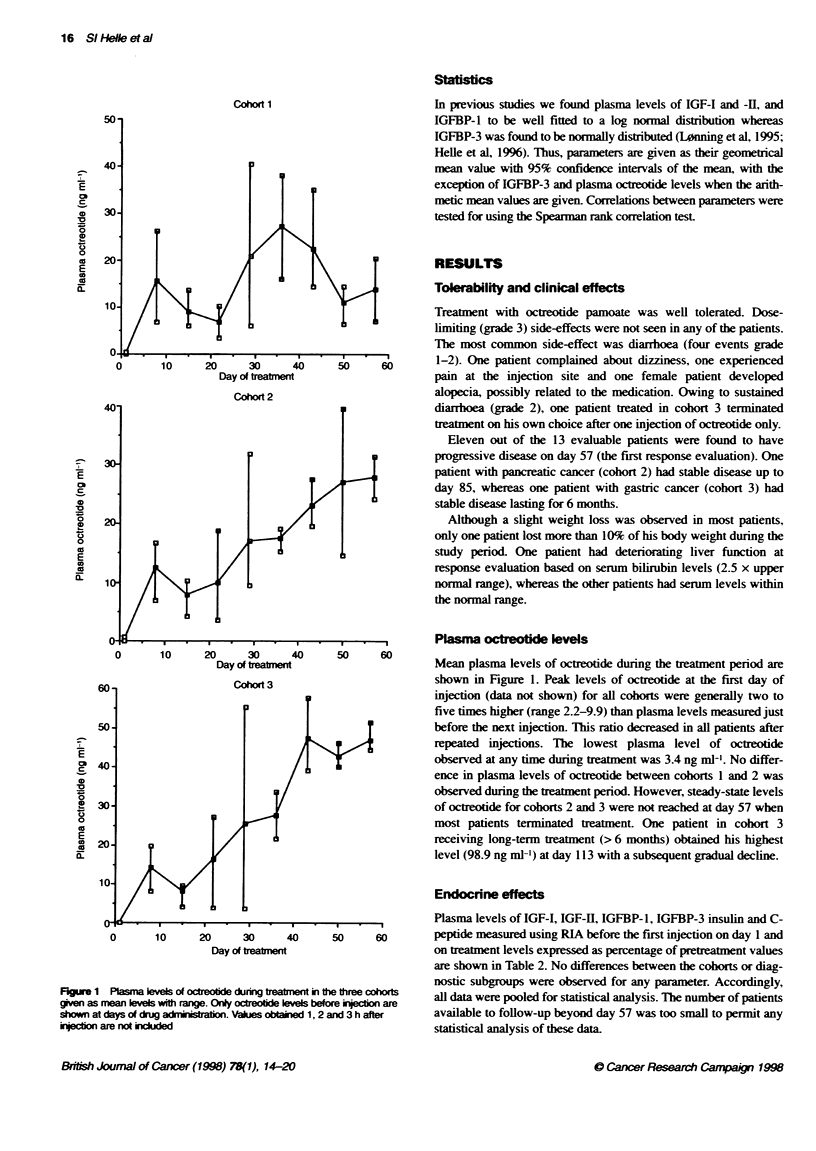

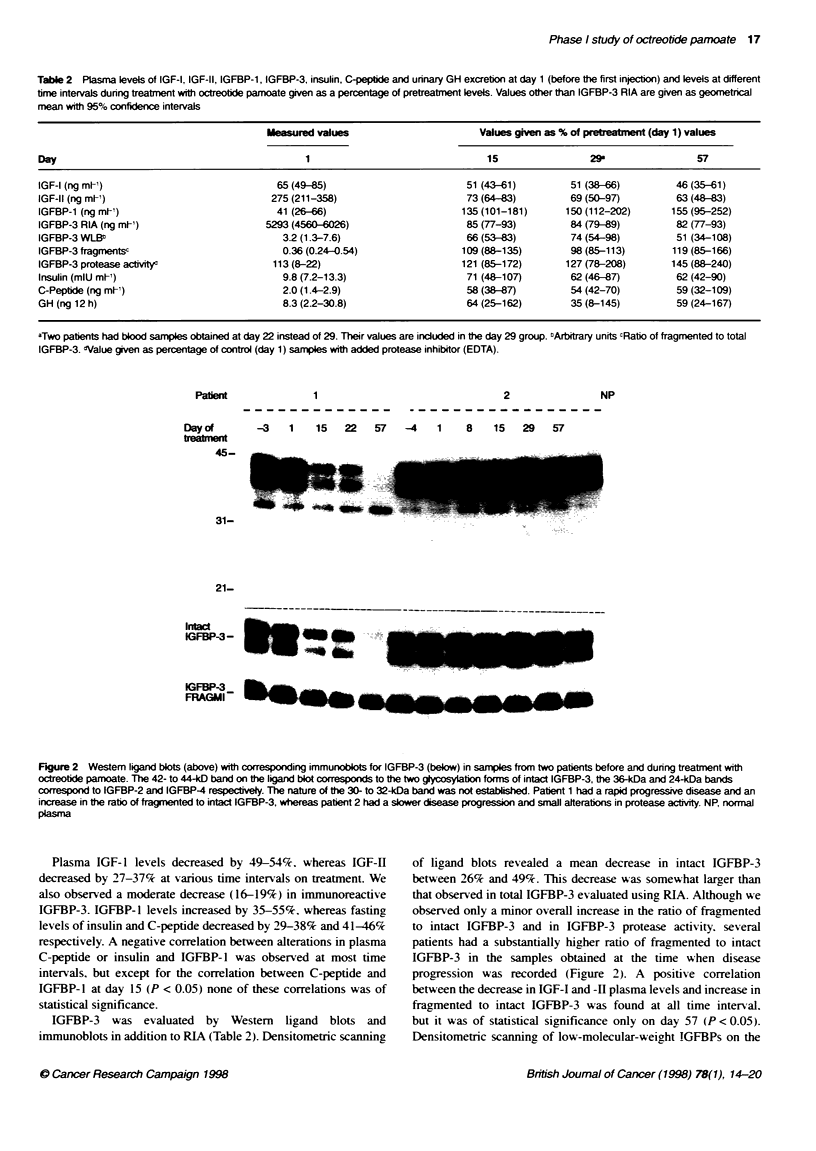

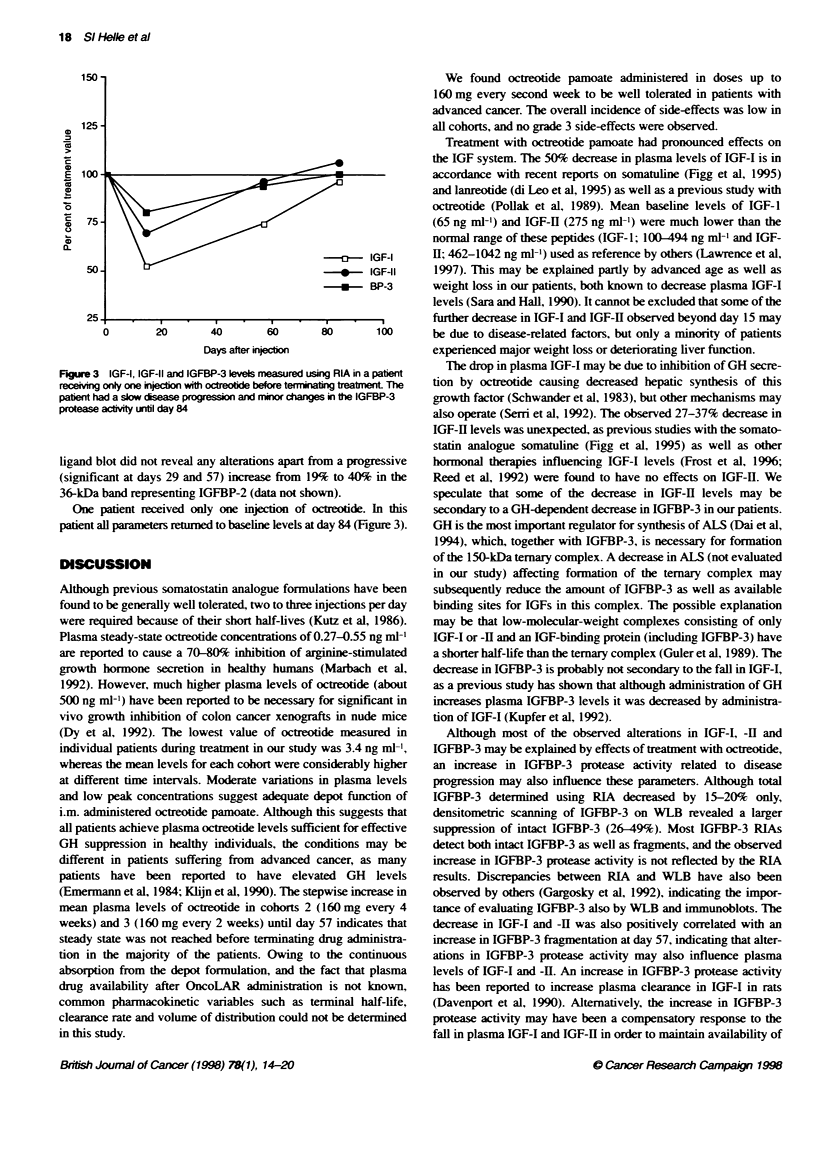

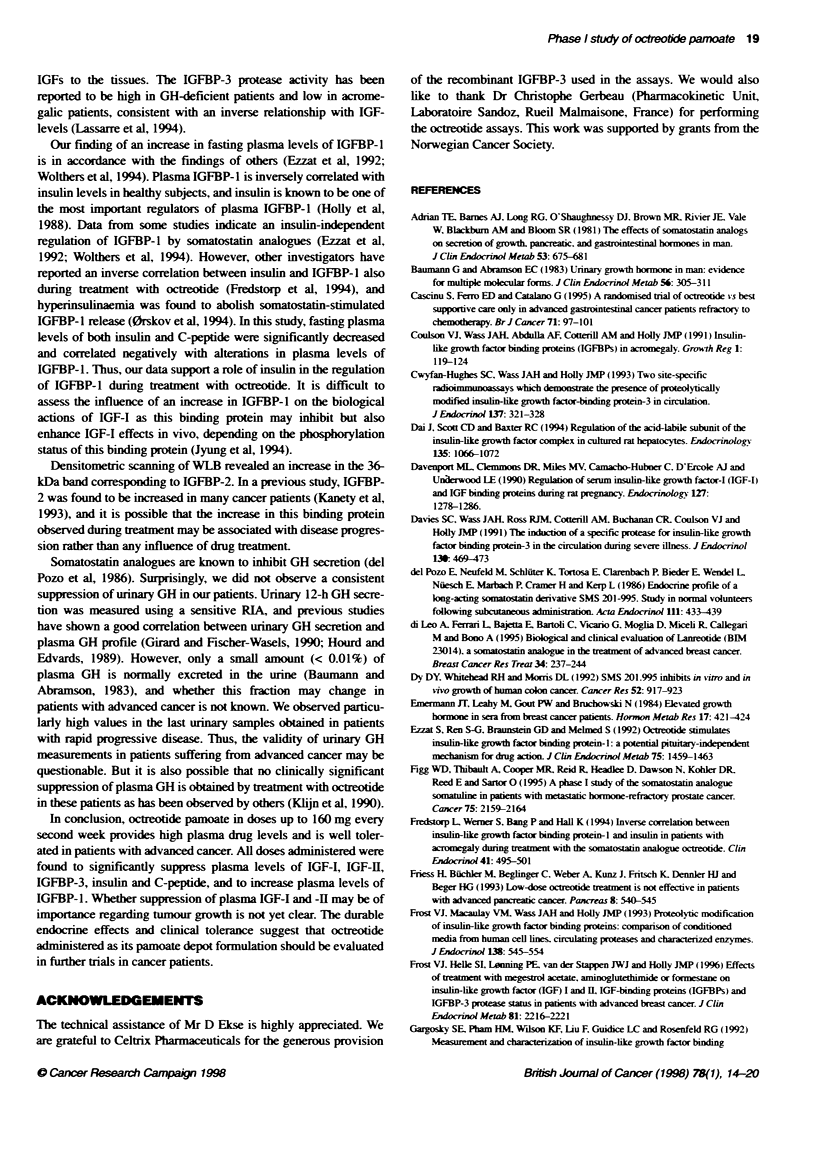

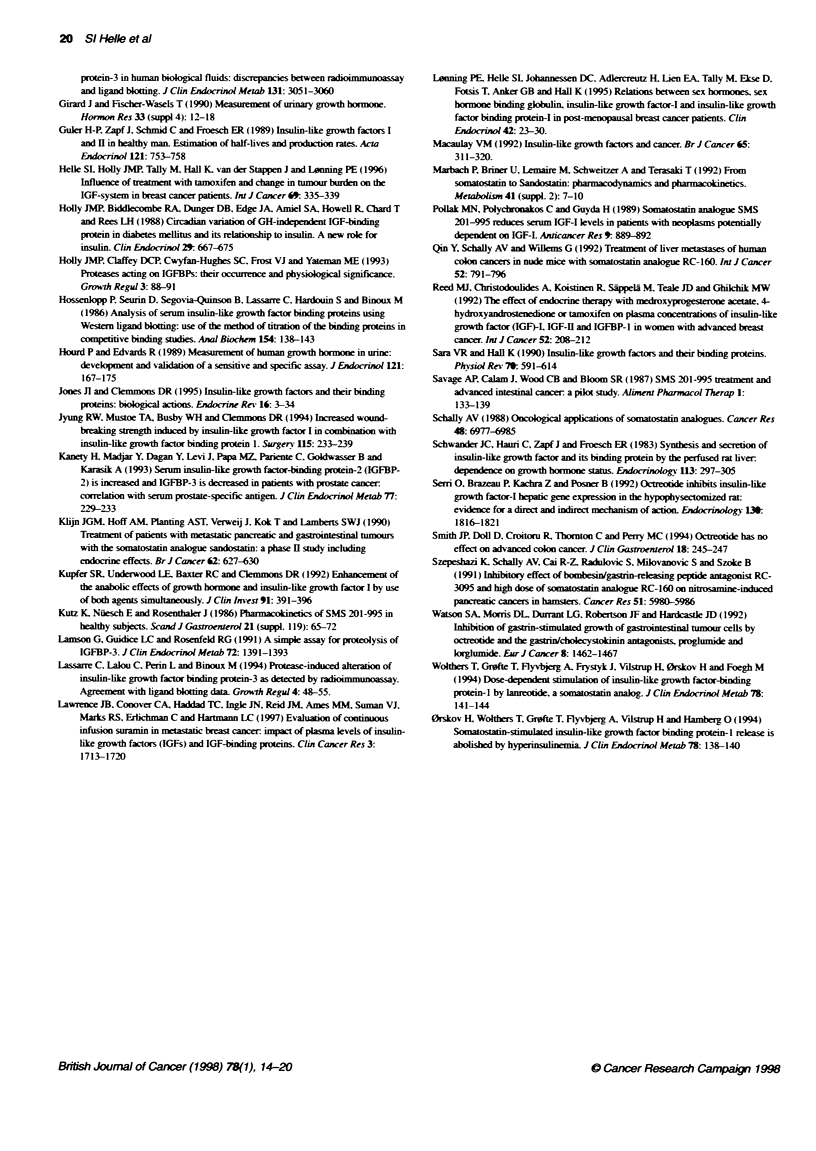


## References

[OCR_00643] Adrian T. E., Barnes A. J., Long R. G., O'Shaughnessy D. J., Brown M. R., Rivier J., Vale W., Blackburn A. M., Bloom S. R. (1981). The effect of somatostatin analogs on secretion of growth, pancreatic, and gastrointestinal hormones in man.. J Clin Endocrinol Metab.

[OCR_00649] Baumann G., Abramson E. C. (1983). Urinary growth hormone in man: evidence for multiple molecular forms.. J Clin Endocrinol Metab.

[OCR_00653] Cascinu S., Del Ferro E., Catalano G. (1995). A randomised trial of octreotide vs best supportive care only in advanced gastrointestinal cancer patients refractory to chemotherapy.. Br J Cancer.

[OCR_00658] Coulson V. J., Wass J. A., Abdulla A. F., Cotterill A. M., Holly J. M. (1991). Insulin-like growth factor binding proteins (IGFBPs) in acromegaly.. Growth Regul.

[OCR_00663] Cwyfan Hughes S. C., Wass J. A., Holly J. M. (1993). Two site-specific radioimmunoassays which demonstrate the presence of proteolytically modified insulin-like growth factor-binding protein-3 in the circulation.. J Endocrinol.

[OCR_00669] Dai J., Scott C. D., Baxter R. C. (1994). Regulation of the acid-labile subunit of the insulin-like growth factor complex in cultured rat hepatocytes.. Endocrinology.

[OCR_00674] Davenport M. L., Clemmons D. R., Miles M. V., Camacho-Hubner C., D'Ercole A. J., Underwood L. E. (1990). Regulation of serum insulin-like growth factor-I (IGF-I) and IGF binding proteins during rat pregnancy.. Endocrinology.

[OCR_00683] Davies S. C., Wass J. A., Ross R. J., Cotterill A. M., Buchanan C. R., Coulson V. J., Holly J. M. (1991). The induction of a specific protease for insulin-like growth factor binding protein-3 in the circulation during severe illness.. J Endocrinol.

[OCR_00693] Di Leo A., Ferrari L., Bajetta E., Bartoli C., Vicario G., Moglia D., Miceli R., Callegari M., Bono A. (1995). Biological and clinical evaluation of lanreotide (BIM 23014), a somatostatin analogue, in the treatment of advanced breast cancer. A pilot study by the I.T.M.O. Group. Italian Trials in Medical Oncology.. Breast Cancer Res Treat.

[OCR_00699] Dy D. Y., Whitehead R. H., Morris D. L. (1992). SMS 201.995 inhibits in vitro and in vivo growth of human colon cancer.. Cancer Res.

[OCR_00703] Emerman J. T., Leahy M., Gout P. W., Bruchovsky N. (1985). Elevated growth hormone levels in sera from breast cancer patients.. Horm Metab Res.

[OCR_00706] Ezzat S., Ren S. G., Braunstein G. D., Melmed S. (1992). Octreotide stimulates insulin-like growth factor-binding protein-1: a potential pituitary-independent mechanism for drug action.. J Clin Endocrinol Metab.

[OCR_00713] Figg W. D., Thibault A., Cooper M. R., Reid R., Headlee D., Dawson N., Kohler D. R., Reed E., Sartor O. (1995). A phase I study of the somatostatin analogue somatuline in patients with metastatic hormone-refractory prostate cancer.. Cancer.

[OCR_00718] Fredstorp L., Werner S., Bang P., Hall K. (1994). Inverse correlation between insulin-like growth factor binding protein-1 and insulin in patients with acromegaly during treatment with the somatostatin analogue octreotide.. Clin Endocrinol (Oxf).

[OCR_00727] Friess H., Büchler M., Beglinger C., Weber A., Kunz J., Fritsch K., Dennler H. J., Beger H. G. (1993). Low-dose octreotide treatment is not effective in patients with advanced pancreatic cancer.. Pancreas.

[OCR_00737] Frost V. J., Helle S. I., Lønning P. E., van der Stappen J. W., Holly J. M. (1996). Effects of treatment with megestrol acetate, aminoglutethimide, or formestane on insulin-like growth factor (IGF) I and II, IGF-binding proteins (IGFBPs), and IGFBP-3 protease status in patients with advanced breast cancer.. J Clin Endocrinol Metab.

[OCR_00732] Frost V. J., Macaulay V. M., Wass J. A., Holly J. M. (1993). Proteolytic modification of insulin-like growth factor-binding proteins: comparison of conditioned media from human cell lines, circulating proteases and characterized enzymes.. J Endocrinol.

[OCR_00756] Girard J., Fischer-Wasels T. (1990). Measurement of urinary growth hormone. A noninvasive method to assess the 'growth hormone status'.. Horm Res.

[OCR_00760] Guler H. P., Zapf J., Schmid C., Froesch E. R. (1989). Insulin-like growth factors I and II in healthy man. Estimations of half-lives and production rates.. Acta Endocrinol (Copenh).

[OCR_00765] Helle S. I., Holly J. M., Tally M., Hall K., Vander Stappen J., Lønning P. E. (1996). Influence of treatment with tamoxifen and change in tumor burden on the IGF-system in breast cancer patients.. Int J Cancer.

[OCR_00772] Holly J. M., Biddlecombe R. A., Dunger D. B., Edge J. A., Amiel S. A., Howell R., Chard T., Rees L. H., Wass J. A. (1988). Circadian variation of GH-independent IGF-binding protein in diabetes mellitus and its relationship to insulin. A new role for insulin?. Clin Endocrinol (Oxf).

[OCR_00776] Holly J. M., Claffey D. C., Cwyfan-Hughes S. C., Frost V. J., Yateman M. E. (1993). Proteases acting on IGFBPs: their occurrence and physiological significance.. Growth Regul.

[OCR_00781] Hossenlopp P., Seurin D., Segovia-Quinson B., Hardouin S., Binoux M. (1986). Analysis of serum insulin-like growth factor binding proteins using western blotting: use of the method for titration of the binding proteins and competitive binding studies.. Anal Biochem.

[OCR_00788] Hourd P., Edwards R. (1989). Measurement of human growth hormone in urine: development and validation of a sensitive and specific assay.. J Endocrinol.

[OCR_00793] Jones J. I., Clemmons D. R. (1995). Insulin-like growth factors and their binding proteins: biological actions.. Endocr Rev.

[OCR_00797] Jyung R. W., Mustoe J. A., Busby W. H., Clemmons D. R. (1994). Increased wound-breaking strength induced by insulin-like growth factor I in combination with insulin-like growth factor binding protein-1.. Surgery.

[OCR_00804] Kanety H., Madjar Y., Dagan Y., Levi J., Papa M. Z., Pariente C., Goldwasser B., Karasik A. (1993). Serum insulin-like growth factor-binding protein-2 (IGFBP-2) is increased and IGFBP-3 is decreased in patients with prostate cancer: correlation with serum prostate-specific antigen.. J Clin Endocrinol Metab.

[OCR_00810] Klijn J. G., Hoff A. M., Planting A. S., Verweij J., Kok T., Lamberts S. W., Portengen H., Foekens J. A. (1990). Treatment of patients with metastatic pancreatic and gastrointestinal tumours with the somatostatin analogue Sandostatin: a phase II study including endocrine effects.. Br J Cancer.

[OCR_00816] Kupfer S. R., Underwood L. E., Baxter R. C., Clemmons D. R. (1993). Enhancement of the anabolic effects of growth hormone and insulin-like growth factor I by use of both agents simultaneously.. J Clin Invest.

[OCR_00821] Kutz K., Nüesch E., Rosenthaler J. (1986). Pharmacokinetics of SMS 201-995 in healthy subjects.. Scand J Gastroenterol Suppl.

[OCR_00825] Lamson G., Giudice L. C., Rosenfeld R. G. (1991). A simple assay for proteolysis of IGFBP-3.. J Clin Endocrinol Metab.

[OCR_00829] Lassarre C., Lalou C., Perin L., Binoux M. (1994). Protease-induced alteration of insulin-like growth factor binding protein-3 as detected by radioimmunoassay. Agreement with ligand blotting data.. Growth Regul.

[OCR_00836] Lawrence J. B., Conover C. A., Haddad T. C., Ingle J. N., Reid J. M., Ames M. M., Suman V. J., Marks R. S., Erlichman C., Hartmann L. C. (1997). Evaluation of continuous infusion suramin in metastatic breast cancer: impact on plasma levels of insulin-like growth factors (IGFs) and IGF-binding proteins.. Clin Cancer Res.

[OCR_00844] Lønning P. E., Helle S. I., Johannessen D. C., Adlercreutz H., Lien E. A., Tally M., Ekse D., Fotsis T., Anker G. B., Hall K. (1995). Relations between sex hormones, sex hormone binding globulin, insulin-like growth factor-I and insulin-like growth factor binding protein-1 in post-menopausal breast cancer patients.. Clin Endocrinol (Oxf).

[OCR_00852] Macaulay V. M. (1992). Insulin-like growth factors and cancer.. Br J Cancer.

[OCR_00856] Marbach P., Briner U., Lemaire M., Schweitzer A., Terasaki T. (1992). From somatostatin to sandostatin: pharmacodynamics and pharmacokinetics.. Metabolism.

[OCR_00927] Orskov H., Wolthers T., Grøfte T., Flyvbjerg A., Vilstrup H., Hamberg O. (1994). Somatostatin-stimulated insulin-like growth factor binding protein-1 release is abolished by hyperinsulinemia.. J Clin Endocrinol Metab.

[OCR_00871] Reed M. J., Christodoulides A., Koistinen R., Seppälä M., Teale J. D., Ghilchik M. W. (1992). The effect of endocrine therapy with medroxyprogesterone acetate, 4-hydroxyandrostenedione or tamoxifen on plasma concentrations of insulin-like growth factor (IGF)-I, IGF-II and IGFBP-1 in women with advanced breast cancer.. Int J Cancer.

[OCR_00879] Sara V. R., Hall K. (1990). Insulin-like growth factors and their binding proteins.. Physiol Rev.

[OCR_00883] Savage A. P., Calam J., Wood C. B., Bloom S. R. (1987). SMS 201-995 treatment and advanced intestinal cancer: a pilot study.. Aliment Pharmacol Ther.

[OCR_00888] Schally A. V. (1988). Oncological applications of somatostatin analogues.. Cancer Res.

[OCR_00892] Schwander J. C., Hauri C., Zapf J., Froesch E. R. (1983). Synthesis and secretion of insulin-like growth factor and its binding protein by the perfused rat liver: dependence on growth hormone status.. Endocrinology.

[OCR_00897] Serri O., Brazeau P., Kachra Z., Posner B. (1992). Octreotide inhibits insulin-like growth factor-I hepatic gene expression in the hypophysectomized rat: evidence for a direct and indirect mechanism of action.. Endocrinology.

[OCR_00904] Smith J. P., Doll D., Croitoru R., Thornton C., Perry M. C. (1994). Octreotide has no effect on advanced colon cancer.. J Clin Gastroenterol.

[OCR_00908] Szepeshazi K., Schally A. V., Cai R. Z., Radulovic S., Milovanovic S., Szoke B. (1991). Inhibitory effect of bombesin/gastrin-releasing peptide antagonist RC-3095 and high dose of somatostatin analogue RC-160 on nitrosamine-induced pancreatic cancers in hamsters.. Cancer Res.

[OCR_00916] Watson S. A., Morris D. L., Durrant L. G., Robertson J. F., Hardcastle J. D. (1992). Inhibition of gastrin-stimulated growth of gastrointestinal tumour cells by octreotide and the gastrin/cholecystokinin receptor antagonists, proglumide and lorglumide.. Eur J Cancer.

[OCR_00920] Wolthers T., Grøfte T., Flyvbjerg A., Frystyk J., Vilstrup H., Orskov H., Foegh M. (1994). Dose-dependent stimulation of insulin-like growth factor-binding protein-1 by lanreotide, a somatostatin analog.. J Clin Endocrinol Metab.

[OCR_00686] del Pozo E., Neufeld M., Schlüter K., Tortosa F., Clarenbach P., Bieder E., Wendel L., Nüesch E., Marbach P., Cramer H. (1986). Endocrine profile of a long-acting somatostatin derivative SMS 201-995. Study in normal volunteers following subcutaneous administration.. Acta Endocrinol (Copenh).

